# Dengue NS1 antigen as a marker of severe clinical disease

**DOI:** 10.1186/s12879-014-0570-8

**Published:** 2014-10-31

**Authors:** Shiran Ajith Paranavitane, Laksiri Gomes, Achala Kamaladasa, Thiruni N Adikari, Nilanka Wickramasinghe, Chandima Jeewandara, Narangoda Liyanage Ajantha Shyamali, Graham Stuart Ogg, Gathsaurie Neelika Malavige

**Affiliations:** Centre for Dengue Research, Faculty of Medical Sciences, University of Sri Jayawardanapura, Nugegoda, Sri Lanka; Department of Medicine, Faculty of Medical Sciences, University of Sri Jayawardanapura, Nugegoda, Sri Lanka; Department of Dermatology, Churchill Hospital, Oxford, OX3 7LJ UK; MRC Human Immunology Unit, Weatherall Institute of Molecular Medicine, Oxford NIHR Biomedical Research Centre and University of Oxford, Oxford, OX3 9DS UK

**Keywords:** Dengue, Severity prediction, NS1 antigen, Point of care test

## Abstract

**Background:**

Early detection of complications significantly reduces dengue associated mortality and morbidity. We set out to determine if the NS1 rapid antigen detection test could be used as a point of care test to predict severe disease.

**Methods:**

186 adult patients with confirmed dengue were enrolled during day 3-8 of illness. Clinical and laboratory parameters were recorded during the course of the illness and NS1 antigen levels were determined using both the Panbio dengue early ELISA (Panbio, Australia) and a NS1 rapid antigen detection kit (SD Bioline, South Korea).

**Results:**

59.1% of patients presented to hospital on day 5-6 of illness when NS1 antigen positivity was significantly (p = 0.008) associated with severe dengue (odds ratio 3.0, 95% CI 1.39 to 6.47) and the NS1 antigen levels were significantly higher (p = 0.03) in those who went on to develop shock. Serum NS1 antigen levels significantly (p < 0.0001) and inversely correlated with the total white cell counts and lymphocyte counts. The bedside NS1 test showed comparable sensitivity (97.4%) and specificity (93.7%) to the laboratory NS1 test in our setting and cohort.

**Conclusion:**

NS1 antigen positivity is associated with a higher risk of developing severe dengue especially when positive beyond day 5 of illness in our cohort, and while further validation studies are required, the test can therefore potentially be used as a bedside point of care test as a warning sign of severe dengue.

**Electronic supplementary material:**

The online version of this article (doi:10.1186/s12879-014-0570-8) contains supplementary material, which is available to authorized users.

## Background

Dengue infections are currently one of the most rapidly emerging arboviral infections in the world [1], which result in 390 million infections every year [2]. They cause significant morbidity and mortality especially in resource poor developing countries and is a huge burden on their economies [3]. Although the majority of dengue infections result in asymptomatic infection or manifest as undifferentiated viral fever, some develop fluid leakage and bleeding manifestations which result in dengue haemorrhagic fever (DHF) and dengue shock syndrome (DSS) [4]. As there is no effective antiviral treatment or a licensed vaccine to prevent infection, meticulous fluid management and monitoring for complications is currently the only option available.

Earlier case fatalities due to dengue infection have been reported to be around 2.5% to 5.4% [5],[6]. Shock and organ impairment have been shown to be the most important factors that lead to fatalities in dengue infection [7],[8]. As a result of better fluid management regimes and greater awareness of associations of severe dengue and early interventions, the case fatality rates have significantly dropped in many dengue endemic countries [9]. However, in order for early detection of those who are likely to develop severe dengue, the clinical and laboratory parameters are measured at least two or three times a day in all patients admitted to the hospital with dengue infection. Therefore, such intense monitoring of all patients has caused a great strain to resource poor health care facilities in many dengue affected countries. Although optimum management of patients includes monitoring of many clinical parameters at least every 2 hours [3], this is sometimes impossible due to scarce health resources. Therefore, a simple test that can be done in a ward would be of utmost importance to determine the patients who are most likely to develop severe clinical disease.

Detection of the dengue virus by virus isolation or by nucleic acid detection methods are considered as confirmatory tests for confirming the diagnosis of dengue infection [3]. However, due to the need for advanced laboratory facilities these two methods may not be suitable for routine diagnosis of dengue virus infection early in the disease in resource poor communities. Many commercial assays are currently available for the detection of dengue NS1 antigen, which is a non structural protein of the dengue virus [10]. Among these tests, the Panbio early dengue NS1 capture ELISA has shown to have an overall sensitivity of 60.4 to 66% and a specificity of 97.9 to 99% [10]-[12]. It has also been shown that NS1 rapid antigen detection tests (SD Diagnostics, Bioline, South Korea) has a [13] sensitivity of 81.6% and a specificity of 92% [14].

Dengue NS1 antigen levels have been shown to associate with disease severity and levels have shown to be higher in those with DHF when those with dengue fever [15]. NS1 levels were analysed in 32 children, in whom NS1 antigen levels of >600 ng/ml within the first 72 hours has a sensitivity of 72% and a specificity of 79% in identifying those who are likely to develop DHF [15]. However, performing laboratory NS1 antigen levels in all patients admitted to hospitals in resource poor countries can be challenging, and the majority of adults present to hospital in day 5 of illness [16]. Therefore, we proceeded to determine whether a bedside NS1 rapid antigen detection test could be used as a point of care test in predicting those who are likely to develop severe dengue.

In this study we evaluated the usefulness of the NS1 antigen detection test in predicting the development of severe dengue in adult patients with acute dengue infection. We have used the commercial Panbio early ELISA NS1 antigen detection kit which requires laboratory facilities for generation of results and compared it with a NS1 rapid antigen detection kit (SD Diagnostics, South Korea) which can be used at the bedside of a patient.

## Methods

### Patients

The study was carried out in the year 2013, at the Colombo South Teaching Hospital, which is a tertiary care hospital in Colombo with a bed strength of over 1000. 186 adult patients, who were admitted with a suspected acute dengue infection, were recruited following informed written consent. The study was approved by the Ethics Review Committee of the Faculty of Medical Sciences University of Sri Jayawardenapura. All patients who were aged over 18 years, with features of a possible dengue infection were included following written consent. Those who had a febrile illness due to other infections such as pneumonia, urinary tract infection were excluded. All clinical features such as fever, blood pressure, presence of any bleeding manifestations and presence of any possible fluid accumulation in the pleural cavity and abdomen were monitored several times a day from the time of admission to hospital, until they were discharged. Bleeding manifestations were defined as the presence of petechiae, ecchymoses, epistaxis, haematemesis, melaena or the presence of per vaginal bleeding in the absence of the monthly period in women. Serial recordings of laboratory investigations such as full blood counts were made for entire duration of the illness. Serum alanine transaminase (ALT) and aspartate transaminase (AST) levels were performed on admission in all patients. Based on the 2011 WHO diagnostic criteria, shock was defined as lowering of pulse pressure to 20 mmHg or less or the presence of signs of poor capillary perfusion (cold extremities, poor capillary refill or a rapid pulse rate) [3]. None of the patients had shock before admission or during admission.

In order to determine the usefulness of the NS1 antigen positivity as a marker of severe disease we classified severe disease if the patients had any one of the following clinical or laboratory features. These were evidence of fluid leakage (clinical and/or radiological or a rise in haematocrit of >20% of the baseline) or a narrow pulse pressure (>20 mmHg) or platelet count of <25000 cells/mm^3^ or liver enzyme levels of >500 IU or the presence of bleeding manifestations or any evidence of myocarditis or encephalopathy. Those who did not have even one of the above criteria were categorized to the non-severe dengue group.

### Serology

Acute dengue infection was confirmed by testing the serum samples which were collected on the day of admission with the early dengue NS1 capture ELISA (Panbio, Brisbane, Australia) or in those who were negative for NS1 antigen by using a commercial capture-IgM and IgG ELISA (Panbio, Brisbane, Australia). In order to compare the results of dengue NS1 antigen early ELISA (Panbio, Brisbane, Australia) we also performed the NS1 antigen detection test with a NS1 rapid test according to manufacturer's instructions (SD Bioline). Positive and negative controls were included as part of the assessment. The NS1 rapid antigen tests were carried out on the day of admission and the results were made available to the medical staff caring for the patients.

### Quantitative IL-10 cytokine assays

Quantitative cytokine assays were done in duplicate on serum according to manufacturer's instructions. Serum IL-10 levels (Mabtech, Sweden) were done in 107 patients at the time of admission.

### Statistical analysis

Statistical analysis was performed using Graphpad PRISM version 6. As the data were not normally distributed, differences in the two groups were compared using the Mann-Whitney U test (two tailed). The results were expressed as the median and the inter quartile range (IQR). Degree of association between clinical parameters and disease severity was expressed as the odds ratio (OR), which was obtained from standard contingency table analysis by Haldane's modification of Woolf's method. Degree of association between clinical parameters and NS1 levels were done using Spearman's correlation. The positive predictive value and the negative predictive values were also calculated by using this table. The Fisher's exact test was used to determine the p value. Receiver-operator characteristic (ROC) curves showing the area under the curve (AUC) were generated to determine the discriminatory performance of NS1 antigen levels (Panbio ELISA units) between those with severe dengue and non severe dengue.

## Results

Of the 186 patients, 94 (50.5%) had severe dengue and 92 (49.5%) had non severe dengue based on our disease classification. Patients with severe dengue presented to hospital on an average of day 5.04 (SD ± 1.12) of illness and those with non severe dengue presented on an average of day 5.01 (SD ± 1.11) of illness. The clinical features of these patients are shown in Table 1. Those with evidence of fluid leakage, bleeding manifestations, pulse pressure (difference between systolic and diastolic pressure) of ≤20 mmHg, platelet counts of <25,000 cells/mm^3^ or with liver enzymes >500 IU (>12 times the upper limit of normal range) are at a significantly higher risk of developing severe dengue and should be very closely monitored. Therefore, in order to determine the usefulness of using NS1 as a predictor of severe clinical disease we classified patients as having severe dengue if they had one or more of the above described clinical or laboratory features.

**Table 1:** **Clinical and laboratory features of patients with DF and DHF**

Clinical feature	Severe dengue	Non severe dengue	P value
	N = 94 (%)	N = 92 (%)	
	Median (IQR)^#^	Median (IQR)^#^	
Pleural effusions	25 (26.6)	0	N/A
Ascites	6 (6.4)	0	N/A
Bleeding manifestations	23 (24.5)	0	N/A
Pulse pressure ≤20 mmHg	25 (26.6)	0	N/A
AST levels	165.6 (93.5 to 313.7)	71.6 (45.9 to 128.2)	<0.0001*
ALT levels	106.4 (63.7 to 227.1)	41.4 (27.4 to 108.4)	<0.0001*
Platelet counts	23,000 (15,000 to 42,000)	72,000 (44,250 to 112,000)	<0.0001*
Lowest lymphocytes counts	800 (571.3 to 1223)	1040 (745 to 1648)	0.03*
Lowest neutrophil count	1260 (840 to 1904)	1280 (800 to 1830)	0.93

*Differences in the two groups were compared using the Mann-Whitney U test (two tailed).

^#^Values are for laboratory tests.

### Dengue NSELISA positivity and clinical parameters

**Figure 1:**
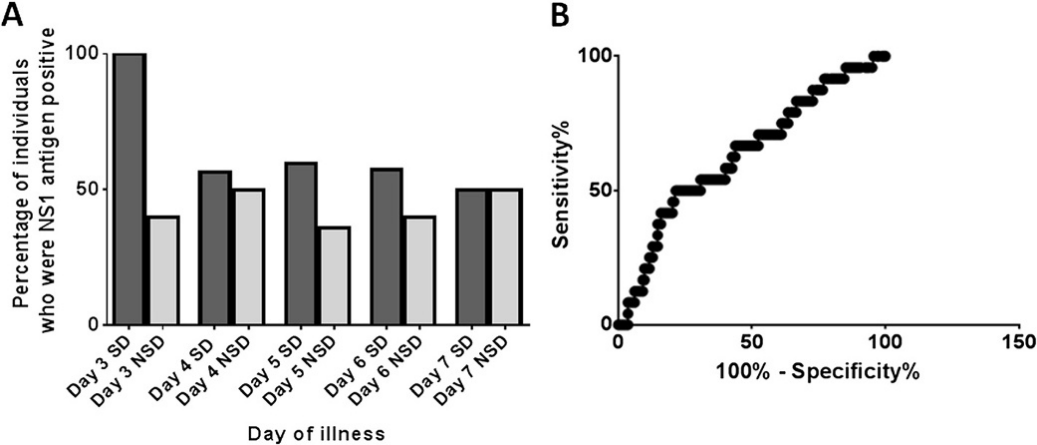
**NS1 antigen positivity in relation to disease severity. A**: Percentage of individuals with severe dengue (SD) and non severe dengue (NSD) who were positive for dengue NS1 antigen detection test by the Panbio NS1 early ELISA. The dark bars represent patients with severe dengue and the lighter bars represent patients with non severe dengue. **B**: The ROC curves of NS1 antigen levels (Panbio units) as a predictor of shock

Based on the above disease classification criteria, 52 (55.3%) with severe dengue and 46 (50%) with non severe dengue were positive for NS1 antigen at the time of admission which was not significantly different. 6/9 (66.7%) were positive for NS1 Ag on day 3 of illness, 23/43 (53.5%) on day 4, 45/94 (47.9%) on day 5, 8/17 (47 %) on day 6 and 5/13 (38.4 %) on day 7 and 4/7 (57.1%) on day 8. Patients who proceeded to develop severe dengue were more likely to have a positive NS1 antigen detection test especially during day 5 to 6 of illness (Figure 1A). 35 (63.6%) of those with severe dengue were positive for NS1 Ag between day 5-6 whereas 21 (36.8%) of those with non severe dengue were also positive. Therefore, at the time of typical first presentation to hospital (between day 5 to 6 of illness) NS1 antigen positivity was significantly (p = 0.008) associated with severe dengue (odds ratio 3.0, 95% CI 1.39 to 6.47). The positive predictive value of a NS1 antigen positivity between days 5-6 of illness and development of severe clinical disease was 63.6 (95% confidence interval 49.6 to 76.1) and the negative predictive value was 63.2 (95% confidence interval 49.3 to 75.5).

A total of 25 (13.4%) developed shock as defined by having a pulse pressure of ≤20mmHg at some point of their illness. The NS1 antigen test (Panbio ELISA) was positive in 16 (64%) of those with shock when compared to those 76 (47.2%) who did not develop shock. The NS1 antigen levels were significantly higher (p = 0.03) in those who went on to develop shock (median 44.9, IQR was 1.7 to 55.4 Panbio units), when compared to those who did not develop shock (median 5.01, IQR was 0.96 to 46.98 Panbio units). Although not significant (p = 0.14) NS1 positivity was associated with a higher likelihood of developing shock (odds ratio = 1.98, 95% CI 0.83 to 4.8, likelihood ratio = 1.8). The NS1 antigen positivity was associated with a 90.4% specificity for developing shock but a very low sensitivity (17.4%). However, NS1 antigen levels at the time of admission did not have a good discriminatory value in predicting those who are likely to develop shock as the area under the receiver operator curve (ROC) curve was 0.63 (95% CI 0.51 to 0.75) (Figure 1B). NS1 antigen levels of >48.49 (Panbio units) at the time of admission were associated with a specificity of 80.25% (73.27 to 86.08%) and a sensitivity of 41.67 % (22.11 to 63.36%) of developing shock.

### Dengue NSELISA positivity and laboratory parameters

**Figure 2:**
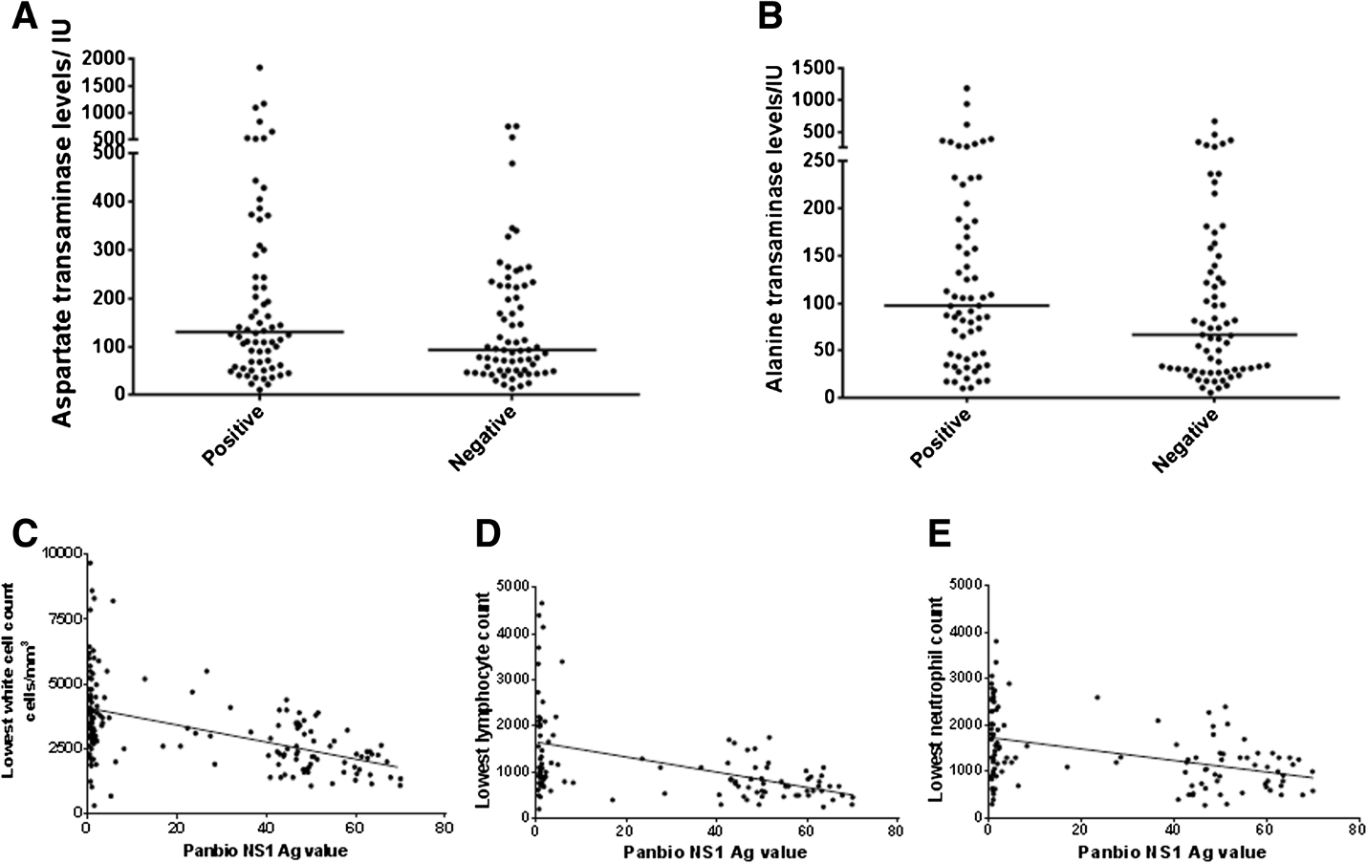
**Dengue NS1 positivity and severity of clinical disease. A**: Aspartate transaminase levels in patients who were NS1 antigen positive and also of those who were NS1 antigen negative at the time of admission. The bar represents the mean. **B**: Alanine transaminase levels in patients who were NS1 antigen positive and also of those who were NS1 antigen negative at the time of admission. The bar represents the mean. **C**: Correlation of white cell counts of patients with dengue infection with NS1 antigen levels (Panbio units). p < 0.0001, Spearman's R: -0.52. **D**: Correlation of the lowest lymphocytes observed during the course of the illness in patients with dengue infection with NS1 antigen levels (Panbio units). p < 0.0001, Spearman's R = -0.48. **E**: Correlation of the lowest neutrophils observed during the course of the illness in patients with dengue infection with NS1 antigen levels (Panbio units). p < 0.0001, Spearman's R = -0.39.

Although not significant (p = 0.059), those who went on to develop severe dengue had higher NS1 antigen levels (Panbio units) on day of admission (median 28.02, IQR 1.01 to 51.24 Panbio units) when compared to those who did not develop severe disease (median 2.9, IQR 0.97 to 44.69 Panbio units). Those who were NS1 antigen positive on admission had higher (p = 0.1) AST values (median 130.6, IQR 10.9 to 297.7 IU) than NS1 negative patients (median 93.6, IQR 50.9 to 226.5 IU) but this was not significantly different (Figure 2A). Those who were NS1 antigen positive on admission also had higher (p = 0.05) ALT levels (median 97.5, IQR 45.05 to 187.5 IU) when compared to those who were NS1 negative (median 66.8, IQR 30 to 144.8 IU) (Figure 2B).

Progressive leucopenia and rapid decline in platelet counts is known to precede plasma leakage [17] and our previous studies and others have shown that leucopenia and especially lymphopenia was associated with severe dengue [18]-[20]. Therefore, we analysed the relationship between serum NS1 levels and white cell counts. Serum NS1 Ag levels inversely correlated with the total white cell counts (p < 0.0001, Spearman's R: -0.52, Figure 2C), the lowest lymphocyte counts (p < 0.0001, Spearman's R = -0.48, Figure 2D) and lowest neutrophil counts (p < 0.0001, Spearman's R = -0.39 Figure 2E).

In our earlier studies we found that serum IL-10 levels were associated with severe dengue. Therefore, we investigated the relationship between serum IL-10 levels and serum NS1 antigen levels at the time of admission in 89 patients of this cohort. We found that NS1 antigen levels (Panbio units) significantly (p = 0.02) correlated with serum IL-10 levels at the time of admission (Spearmans r = 0.22).

### Comparison of the commercial NS1 capture ELISA (Panbio) with NS1 antigen rapid test (SD)

The NS1 antigen capture ELISA has shown to be specific and sensitive for detection of dengue infection during day of illness. Therefore, we went on to compare the NS1 antigen detection by the capture ELISA (Panbio) with a commercial rapid test (SD, Bioline) which can be done using whole blood at the bedside. Of the 186 patients included in our study NS1 rapid antigen detection test was only performed in 156 patients. Of these 156 patients the NS1 antigen test was positive in 74/76 patients who were NS1 antigen positive by the Panbio ELISA. However, the NS1 rapid antigen test was positive in an additional 5 patients who were negative for dengue NS1 antigen by the Panbio NS1 capture ELISA. Therefore, these results show that NS1 detection NS1 rapid antigen detection test (SD Bioline, South Korea) correlated well with the results of the NS1 ELISA (p < 0.0001). The sensitivity of the NS1 rapid antigen test when compared to the NS1 ELISA was 97.4% (95% CI 90.1% to 99.7%) and the specificity was 93.7% (86.7% to 97.9%) in this setting. Therefore, these results suggest that the NS1 rapid antigen detection test (SD Bioline, South Korea) had a comparable sensitivity and specificity as the Panbio commercial capture NS1 antigen detection ELISA in our cohort.

## Discussion

In this study we have evaluated the use of the NS1 antigen test as a marker of severe dengue infection. We found that NS1 antigen positivity especially beyond day 5 of illness, was associated with a higher risk of developing severe dengue (odds ratio 3.0). In this study in order to evaluate the usefulness of the NS1 antigen positivity as a marker of severe clinical disease, we have used different criteria for the definition of severe clinical disease. This is due to the fact that many patients with an acute dengue infection who has evidence of fluid leakage, bleeding manifestations, platelet counts of <25,000 cells/mm^3^ or liver transaminase levels of >12 times the upper limit of normal are very much at a higher risk of developing shock or organ impairment. In our study the NS1 antigen was positive in 64% of those who went on to develop shock on date of admission when compared to those who did not develop shock (47.2%).

Many have investigated the usefulness of liver transaminase levels, platelet counts and other clinical and laboratory parameters in predicting severe dengue, which have shown that none of these parameters can be used alone to predict severe dengue [21]-[24]. We also found that liver transaminase levels were higher in patients with a positive NS1 antigen test and also that the NS1 antigen levels significantly and inversely correlated with all white blood cell parameters. However, Ju et al. have suggested that platelet and lymphocyte counts along with serum IL-10 levels were the most important variables associated with severe dengue [25]. Duen et al. have shown that higher NS1 antigen levels on day 3 of infection were associated with lower platelet counts although they have not correlated the kinetics of NS1 antigen levels with the overall clinical disease severity [26]. Libraty et al. have undertaken kinetics of NS1 levels in acute dengue infection and have shown that NS1 levels were higher in patients with DHF throughout the illness. They have also shown that NS1 levels of >600 ng/ml within the first 72 hours has a sensitivity of 72% and a specificity of 79% in identifying those who are likely to develop DHF [15]. Although we too found that platelet counts were lower in those who were NS1 antigen positive at the time of admission, this was not significant. However, interestingly serum NS1 antigen levels significantly correlated with serum IL-10 levels which is suggested as a possible marker of severe clinical disease [16],[27],[28].

Our previous studies and studies done by others have shown that marked lymphopenia was associated with severe dengue [18],[29]. Since in this study the NS1 antigen levels significantly and inversely correlate with lymphocyte counts (p < 0.0001, R = -0.48), it appears that dengue viraemia could be possibly leading to lymphopenia. Massive T cell apoptosis has been shown to occur in dengue infection, which has been associated with more severe disease [20],[30]. Although the cause of massive T cell death is not clear it has been thought that T cells undergo apoptosis due to massive proliferation and activation induced cell death [31]. Apoptosis of T cells is also shown to reduce following clearance of the virus [31]. Therefore, it is possible that persistent viraemia results in severe clinical disease by facilitating T cell apoptosis either directly or either due to the host response to the virus. However, since it has been shown that dengue virus specific T cells are absent or present in very low numbers in acute dengue infection [31]-[33], and also that dengue virus specific T cell responses are inhibited by immunosuppressive cytokines [32], it is possible that prolonged viraemia leads to severe clinical disease by directly or indirectly inhibiting T cells.

## Conclusion

In our study, we have compared the Panbio early NS1 antigen capture ELISA with a NS1 rapid antigen detection test (SD Bioline, South Korea). While the former requires a well-equipped laboratory, the NS1 rapid antigen detection test can be performed using whole blood at the bedside or in an outpatient department. Our results are similar to others which have shown that the rapid NS1 antigen detection test is of comparable sensitivity and specificity to the NS1 antigen capture ELISA [14]. Although further validation studies are required, our data suggest that the NS1 antigen detection rapid test can potentially be used as a simple investigation which could contribute to warning signs of development of possible severe dengue.

## Authors' contributions

GNM and GSO were involved in study design, analysis of data and writing the manuscript. LG, SAP, AK, NW, CJ and TA collected data and carried out the experiments. NLAS was involved in study design and analysis of data. All authors read and approved the final manuscript.

## Authors’ original submitted files for images

Below are the links to the authors’ original submitted files for images. Authors’ original file for figure 1 Authors’ original file for figure 2
