# Supplementary figures and images for: Dengue NS1 antigen as a marker of severe clinical disease
Source: BMC Infect Dis. 2014 Oct 31;14:570. doi: 10.1186/s12879-014-0570-8 (PMC4222370; doi:10.1186/s12879-014-0570-8)

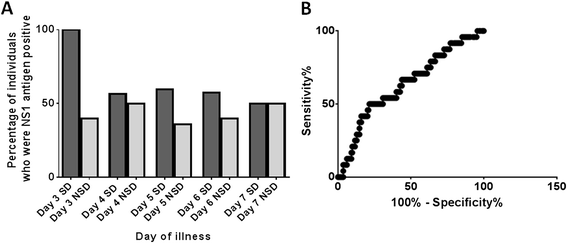

Supplement: Supplementary file 1 — Authors’ original file for figure 1 [file 12879_2014_570_MOESM1_ESM.gif]

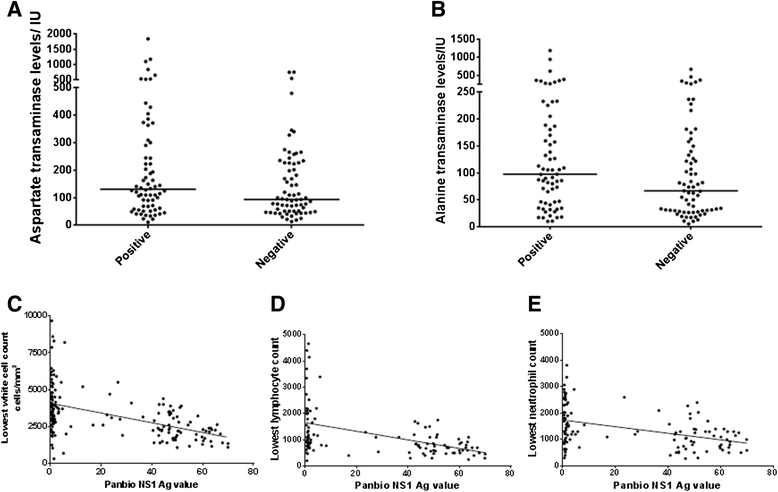

Supplement: Supplementary file 2 — Authors’ original file for figure 2 [file 12879_2014_570_MOESM2_ESM.gif]
